# Earth Microbiome Project and Global Systems Biology

**DOI:** 10.1128/mSystems.00217-17

**Published:** 2018-04-10

**Authors:** Jack A. Gilbert, Janet K. Jansson, Rob Knight

**Affiliations:** a Biosciences Division, Argonne National Laboratory, Argonne, Illinois, USA; b Microbiome Center, Department of Surgery, University of Chicago, Chicago, Illinois, USA; c Earth and Biological Sciences Directorate, Pacific Northwest National Laboratory, Richland, Washington, USA; d Department of Pediatrics, University of California San Diego, La Jolla, California, USA; e Department of Computer Science & Engineering, University of California San Diego, La Jolla, California, USA; f Center for Microbiome Innovation, University of California San Diego, La Jolla, California, USA

**Keywords:** amplicon, global, meta-analysis, metadata, microbiome, systems biology

## EDITORIAL

Recently, we published the first large-scale analysis of data from the Earth Microbiome Project (EMP) (1, 2), a truly multidisciplinary research program involving more than 500 scientists and 27,751 samples acquired from 43 countries. These samples represent myriad specimen types and span a wide range of biotic and abiotic factors, geographic locations, and physicochemical properties. The database (https://qiita.ucsd.edu/emp/) is still growing, with more than 100,000 amplicon data sets and more than 500 paired metagenomic and metabolomic data sets. Importantly, the techniques, data, and analytical tools are all standardized and publicly accessible, providing a framework to support research at a scale of integration that just 7 years ago seemed impossible.

**FIG 1:**
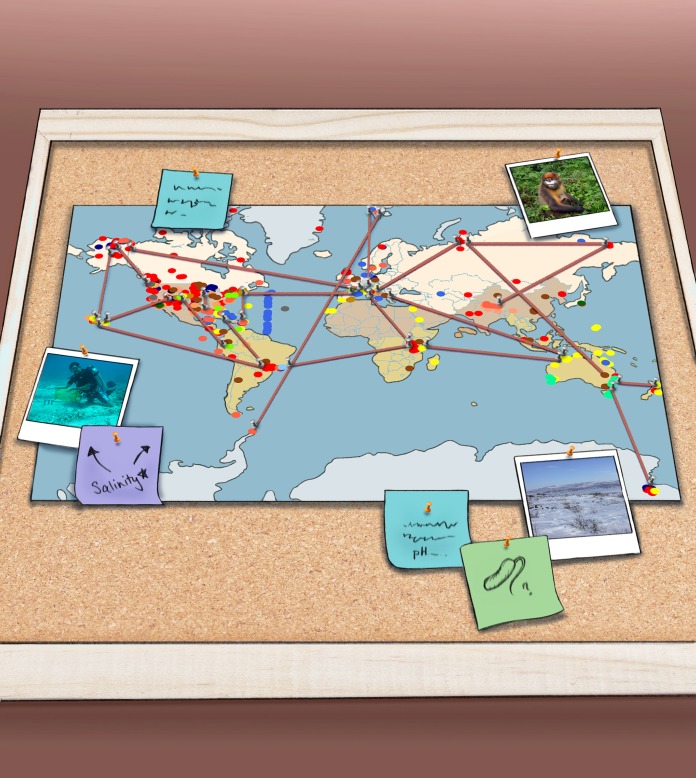
The Earth Microbiome Project was a result of collaborations with hundreds of scientists around the planet; each with their own research interests.

The project started in 2010, as all good things start, with friends and colleagues meeting to discuss ideas. The Terabase Metagenomics meeting was the brainchild of Rick Stevens from Argonne National Laboratory (3) and provided a platform for brainstorming about approaches that would be necessary to create a database of the microbially diverse populations of the planet. Our goal was that the database would be publically accessible, driven by community engagement, and be able to test fundamental hypotheses around microbial ecology. The community responded. Researchers from around the world were willing to share their samples and their associated metadata (sample and site information), so that the EMP could process material and data through a standardized pipeline (Fig. 1).

Standardization allowed disparate samples to be compared with respect to their microbial compositions and diversity. Before the EMP, this was simply impossible, because different investigators used different DNA extraction methods, sequencing protocols, and analytical tools that had been established over years and optimized for their specific sample types. For example, hundreds of different protocols have been developed for extraction of DNA from soil, each with their own biases (4). This presented a major impediment for cross-sample, cross-site, and cross-study comparisons. The diversity of sample types included in the EMP ranged from soil samples, to human and animal stools and skin samples, to marine and freshwater samples. The EMP even went to extremes to try to collect samples not commonly represented in sequence databases to extend our knowledge of the associated microbiota: examples include bird eggshells, ants, bats, polluted marine sediments, and soil samples from deserts and Antarctica and North American permafrost. The three main labs that established the EMP overcame this hurdle by establishing common protocols for DNA extraction, used the same primers and sequencing protocols—and even performed the DNA extractions and sequencing in defined facilities (to overcome known bias between sequencing facilities). In addition, we established QIIME as a common analytical framework and Qiita as a unified database for data and metadata storage. We also established a common metadata platform and required all EMP investigators to provide their metadata before the samples could be sent for DNA extraction and sequencing, greatly facilitating analysis.

The EMP has from the start been challenged to justify this approach and to justify the massive effort required to create this database. The primary justification for this effort is advancing scientific knowledge. We considered but rejected the concept of sampling the earth on a grid, sampling randomly, or sampling with a stratified sampling design, on the grounds that an up-front investment of billions of dollars would be required to do it correctly (although such an investment would be immensely valuable and comparable to existing efforts in earth observation such as satellites). Instead, hypothesis-driven research was an essential part of the EMP from day 1. The EMP is essentially a resource that provides a framework for comparative analysis, so that individual hypothesis-driven studies can be combined to test hypotheses that require bigger data sets or data from a wider range of environments or need to be replicated in multiple systems to establish generality. Therefore, the EMP is a meta-analysis resource that leverages individual hypothesis-driven experiments to ask new questions. As a result, >50 published studies have already generated data through the EMP (http://www.earthmicrobiome.org/publications/). These publications represent individual studies from myriad ecosystems, including the microbiota associated with the digestive system of carnivorous pitcher plants (5), time series studies of the dynamics of freshwater lake microbiota (6), the microbiota of saliva in mice who drink wine (lean and obese men) (7), and the microbiota of permafrost soils from boreal forests in Alaska (8). Many large consortia also worked with the EMP to derive data that could aid in the development and testing of many novel hypotheses, such as the phylogenetic distribution of microbiota across sponges around the world (9). These studies underpin the meta-analysis that was recently published (2).

So what did this meta-analysis uncover? First and foremost, the volume and environmental distribution of these data enabled us to test fundamental hypotheses in biogeography, including determining patterns that have previously been possible only for “macrobial” ecology. In addition, the ecological trends demonstrated key organizing principles whereby ecosystems with less diversity maintained taxa that were found in samples with greater diversity. This nested ecology extended our understanding of microbial seed banks that support global distribution networks (10, 11). Importantly, the data generated have also been used to explore the factors that underpin global diversity trends (12) and by using informatics techniques that highlight the local adaptation and therefore environmental specificity of subspecies.

So where do we go from here? Although the EMP has provided a tremendous resource to the scientific community, it is focused on the taxonomic representation of the community members. What is lacking is understanding of the functional roles that they carry out. Therefore, the next step will be to use a combination of metagenome sequencing and complementary omics technologies (e.g., metatranscriptomics, metaproteomics, and metabolomics) to determine the functional complement of genes, which genes are expressed and translated into proteins and what metabolic processes are carried out. A multi-omics approach has already been used to evaluate microbiomes in targeted habitats. Examples from our own research include permafrost before and after thaw (13), the deep-sea oil plume (14) and contaminated sediments (15) resulting from the Deepwater Horizon oil spill in the Gulf of Mexico, and the human gut following a resistant starch diet (16). An EMP-type approach is now being planned to standardize multi-omics across sample types in order to glean functional comparisons of microbiomes across the planet. To begin with, 500 EMP samples that cover a variety of diverse sample types have been selected for metagenomics and metabolomics. This next step on the EMP horizon will be to provide the foundation for gaining a mechanistic understanding of the roles carried out by earth’s microbiomes and will allow us to test the limits of our ability to extrapolate from taxonomy to function, as well as to integrate observations into models of microbiome change at different levels.

Additionally, with the amplicon protocols well established, we invite the scientific community to generate their own data using the EMP protocols and contribute the data to the Qiita database at https://qiita.ucsd.edu/. We will provide a streamlined user interface for incorporating these new data sets into the EMP, capturing standardized as well as study-specific metadata, checking them against the wealth of published studies for quality control and potential issues of sampling handling, and adding them to the global catalog of microbial knowledge. This rapidly expanding community resource will be of immense value in understanding the global distribution of microbial life and uncovering new ecological principles as well as confirming the generality of existing ones across spatial and temporal scales.

This resource is therefore of direct relevance to the microbial systems biology community and all researchers involved in the exploration of relationships between the components of a microbial cell or community. A system is a set of connected components forming a complex whole. A systems component, in a microbiological context, can comprise any biological or even physicochemical element that connects to produce the observed characteristics of the system. By defining the coassociation and distribution of microbial taxonomic components across planet earth, we provide a framework that contextualizes the ecology of those elements, which can in turn aid interpretation of their properties in other systems. Demonstrating that a particular taxonomic unit of relevance to a plant-root system is broadly distributed across defined physicochemical gradients provides evidence for the adaptation of that lineage, which can aid interpretation of its ecological dynamics. We hope this resource will benefit the community.
